# BeyondSilos, a Telehealth-Enhanced Integrated Care Model in the Domiciliary Setting for Older Patients: Observational Prospective Cohort Study for Effectiveness and Cost-Effectiveness Assessments

**DOI:** 10.2196/20938

**Published:** 2020-10-06

**Authors:** Jordi Piera-Jiménez, Signe Daugbjerg, Panagiotis Stafylas, Ingo Meyer, Sonja Müller, Leo Lewis, Paolo da Col, Frans Folkvord, Francisco Lupiáñez-Villanueva

**Affiliations:** 1 Open Evidence Research Group Universitat Oberta de Catalunya Barcelona Spain; 2 Department of Research & Development Badalona Serveis Assistencials Badalona Spain; 3 Graduate School of Health Economics and Management Università Cattolica del Sacro Cuore Roma Italy; 4 Medical Research & Innovation (HEALTHINK) Thessaloniki Greece; 5 PMV Research Group Universität zu Köln Köln Germany; 6 Empirica Gesellschaft für Kommunikations und Technologieforschung GmbH Bonn Germany; 7 International Foundation for Integrated Care Oxford United Kingdom; 8 IGEA Hospital Trieste Trieste Italy; 9 Tilburg School of Humanities and Digital Sciences Tilburg University Tilburg Netherlands; 10 Department of Information and Communication Sciences Universitat Oberta de Catalunya Barcelona Spain

**Keywords:** integrated care, telemedicine, telecare, digital health, cost-effectiveness, clinical effectiveness, chronic disease

## Abstract

**Background:**

Information and communication technology may provide domiciliary care programs with continuity of care. However, evidence about the effectiveness and cost-effectiveness of information and communication technology in the context of integrated care models is relatively scarce.

**Objective:**

The objective of our study was to provide evidence on the clinical effectiveness and cost-effectiveness of the BeyondSilos project for patients enrolled in the Badalona city pilot site in Spain.

**Methods:**

A quasi-experimental study was used to assess the cost-effectiveness of information and communication technology–enhanced integration of health and social care, including the third sector (intervention), compared to basic health and social care coordination (comparator). The study was conducted in Badalona between 2015 and 2016. Participants were followed for 8 months.

**Results:**

The study included 198 patients: 98 in the intervention group and 100 in the comparator group. The mean Barthel index remained unchanged in the intervention group (mean change 0.14, 95% CI –4.51 to 4.78; *P*=.95) but decreased in the comparator group (mean change –3.23, 95% CI –5.34 to –1.11; *P*=.003). Instrumental Activities of Daily Living significantly decreased in both groups: mean changes of –0.23 (95% CI –0.44 to –0.02; *P*=.03) and –0.33 (95% CI –0.46 to –0.20; *P*<.001) in the intervention and comparator groups, respectively. No differences were found in the Geriatric Depression Scale (intervention: mean change 0.28, 95% CI –0.44 to 1.01, *P*=.44; comparator: mean change –0.29, 95% CI –0.59 to 0.01, *P*=.06). The intervention showed cost-effectiveness (incremental cost-effectiveness ratio €6505.52, approximately US $7582).

**Conclusions:**

The information and communication technology–enhanced integrated domiciliary care program was cost-effective. The beneficial effects of this approach strongly rely upon the commitment of the professional staff involved.

**Trial Registration:**

ClinicalTrials.gov NCT03111004; http://clinicaltrials.gov/ct2/show/ NCT03111004

## Introduction

### Background

Domiciliary care programs are increasingly used to deliver health care to patients―particularly older patients and those with chronic conditions―who are unable to go to a primary care center due to their medical condition or disability, thus improving their health and functional independence, while reducing hospitalizations [1-4]. Among domiciliary care programs, integrated care models prioritize continuity in the sense that the same care provider supports the patient both at home and the primary care center. However, the need for integration with social care is often undervalued [5]. The relevance of social care is not limited to the role of social workers, but also that of stakeholders in the third sector, which in some areas may strongly contribute to day-to-day welfare of these patients [6].

Regardless of the involvement of stakeholders from the social domain, integrated domiciliary care models face the challenge of being efficient enough to absorb the rapidly rising number of care recipients in this setting, likely prompted by social and demographic shifts [7]. In fact, the current overloaded schedule of primary care teams involved in integrated domiciliary care programs has been already identified as a significant drawback of this care model [8,9].

Among the interventions designed to increase the efficiency of health care systems, the use of information and communication technologies have shown promising results in various areas, including the management of older people with chronic diseases [10-12]. Besides integrating all patient information and facilitating the coordination of the various professionals involved, information and communication technology provides domiciliary care with telemonitoring solutions, which may bring patients and professionals closer [13]. However, the evidence regarding the cost-effectiveness of these solutions in the context of integrated care models is scarce and heterogeneous in terms of quality [7,14,15].

### The BeyondSilos Project

BeyondSilos aimed to promote community-based, independent lives by providing domiciliary care with information and communication technology solutions capable of crossing through domain boundaries that typically separate social and health care providers [16]. One of the key areas of integration (frequently referred as to horizontal integration) was for the common access of all cross-sectorial care teams, including those of the third sector, to telehealth platforms in order to improve coordination and promote continuity of care.

To overcome the traditional boundaries separating social and health care, information and communication technology solutions of the BeyondSilos project went hand-in-hand with innovative organizational designs. This approach was based on the assumption by Urošević and Mitić, who pointed out that “Successful service integration in policy and practice requires both technology innovation and service process innovation being pursued and implemented at the same time [17].” Because information and communication technology–based services are typically delivered within sociotechnical system (ie, organizational frameworks where people interact with technology), their success often depends on the value of people applying technology. Hence, information and communication technology can effectively support well-designed care service delivery processes, but it cannot replace them because of the emotional aspects of physical meetings [18].

The first step in achieving a combined innovation approach was the development of common integrated care pathways that were to be supported by information and communication technology. For this purpose, the project adopted 2 generic service pathways of the SmartCare project which were adapted to fit local context through service process modeling techniques (Multimedia Appendix 1). The first pathway addressed needs for integrated home care during an acute episodes and immediately after hospital discharge. The second pathway was directed toward people needing integrated long-term care (eg, frail patients with multiple comorbidities).

We hypothesized that the provision of information and communication technology–enhanced integrated care services that encompass health and social care in the setting of domiciliary care would improve health outcomes and reduce health system costs. Herein, we report the clinical effectiveness and cost-effectiveness of the BeyondSilos intervention for patients enrolled in the long-term pathway in a Badalona city pilot site (Spain).

## Methods

### Study Design, Setting, and Participants

As part of the BeyondSilos project, an observational prospective cohort study was carried out to assess the implementation of an information and communication technology–enhanced integrated care model in the setting of domiciliary care in *Badalona Serveis Assistencials* (BSA), a public provider of health and social care services to the City Council of Badalona, the most populated suburban area to the north of Barcelona, Spain with a reference population of 433,175 inhabitants. BSA has recently been shifting toward integrated care models [19-30].

In Spain, the health and social care systems are centrally managed by the Ministry of Health, Consumerism, and Social Services, which provides the basic regulations and guidelines. The political control and jurisdiction over the organization and provision of health and social services are transferred to the 17 regional governments (autonomous communities). The health system is based on a Beveridge model, characterized by universal coverage, funded by the government through tax, and delivered by an extensive network of public and private health providers. The regions have the main responsibility for social services provision, together with municipalities [31,32]. Third-sector organizations (voluntary and nonprofit) play an essential role in responding to many and different social needs of the general population that are beyond the reach of the scarce public resources (eg, volunteer care and accompaniment of those at risk of social exclusion and isolation) [33].

Care recipients assessed for eligibility were involved in a domiciliary care program as described by Burgos-Díez et al (study condition) [19] and were recruited among care recipients managed from 6 primary care centers. Centers acting as intervention and comparator were paired 1-to-1 for similar socioeconomic status in their area of influence. To this end, candidate sites were stratified into 3 categories of socioeconomic status of the catchment area (2 primary care centers per category). The information and communication technology–enhanced integrated care model (intervention) was first introduced in 1 center in each category; the remaining centers were used as comparators. The first care recipient was enrolled March 3, 2015, and the last care recipient exited the project October 20, 2016.

### Eligibility Criteria

The main inclusion criteria were age ≥65 years, special health needs due to the presence of chronic diseases (ie, heart failure, stroke, diabetes, or chronic obstructive pulmonary disease plus at least 1 additional chronic disease included in the Charlson Comorbidity Index [34]), and the need for social care based on Barthel Index of Activities of Daily Living [35] and Instrumental Activities of Daily Living. To be assessed for eligibility, patients were not require to have an active internet or mobile contract but had to have reliable 4G coverage at home (required by the telehealth solution provided). Participants with an active cancer or AIDS diagnosis, in a terminal state, those who had undergone an organ transplant, or who were on dialysis before enrolment were excluded from the study.

### Ethics

The study protocol was approved by the Independent Ethics Committee of the *Hospital Germans Trias i Pujol*, and all participants provided informed consent before entering the study.

### Intervention

Participants from both groups received health and social care, integrated through a corporate enterprise resource planner which was used as a facilitator for administrative coordination between BSA and the municipality (ie, management of admissions and discharges). Health and social care information were stored in 2 centralized repositories linked to each other through interoperability. Domiciliary care was coordinated using a homecare department software, which stored the Shared Care Plan, accessible for both health and social care professionals. Based on this Plan, professionals scheduled regular visits or phone contacts with care recipients.

In addition to the aforementioned common resources, participants in the intervention group were provided with a telehealth platform, the Health Insight Solutions Homecare Platform, which included the following components: security sensors (ie, fire and water detectors, behavioral movement sensors, and a cell phone with GPS tracking and fall detection), medical devices (ie, weight scale, blood pressure meter, glucometer, and oximeter), serious games, a personal diary, and a videoconferencing system (Multimedia Appendix 2). The telehealth platform was used by the participants and their close relatives to continuously track their health status following the care plan defined by their formal caregivers. Information collected within the telehealth platform was checked daily by the primary care team responsible for the patient. Exacerbation of health conditions (eg, weight increase over 20% in a 1-week period) and out-of-hours alarms (ie, fall detection, fire, or water leak) automatically triggered an alert (SMS text message) to the team on call. In the intervention group, third-sector care providers had access to basic clinical information (ie, main diagnostics and visits from other professional staff) throughout the Shared Care Plan and provided volunteer accompaniment support to patients at risk of social exclusion.

### Recruitment

Potential study participants were identified in a 2-stage process. The first part of the process was conducted by the Information Systems Department of BSA and consisted of identifying possible candidates through a database search using the inclusion and exclusion criteria. The initial selection process identified 4800 possible candidates receiving both health and social care services. Applying more specific inclusion criteria, such as diagnosis-based specificities, reduced the list to 430 patients. In a second stage, research assistants in each participating center approached the individuals and asked them if they were willing to participate.

### Assessments

The effectiveness of the intervention was evaluated using the Model for Assessment of Telemedicine [36]. Primary outcome measures were related to the health status of study participants and established based on the Barthel index scale, the Instrumental Activities of Daily Living scales [37], and the Geriatric Depression Scale [38]. All questionnaires were collected online by trained researchers using a purpose-designed survey built on an open-source tool (LimeSurvey; Limesurvey GmbH) [39].

Costs were modeled and collected from both a health care and societal perspective using the ASSIST Tool [40] and were estimated in 2016 euros. For the intervention group, 2 types of costs were considered: one-off costs (ie, incurred only at implementation) and recurring costs (ie, costs derived from the service practice).

The health care costs perspective included the assessment of resource utilization and considered all characteristics regarding hospitalization (eg, number of admissions and readmissions, length of hospital stay, and type of admission) and contacts with health and social care professionals (eg, type of professional, number of contacts, and type and setting of the contact). For personnel costs, the average income for 1 full‐time employee with employer contributions to social security was used. The average hourly wages were €29.23 for a physician (approximately US $34.07), €20.79 for a nurse (approximately US $24.23), and €18.19 for a social care worker (approximately US $21.20).

The societal cost perspectives considered were the health care costs plus those outside the health care sector. In this case, the costs for the intervention group included the time spent by patients using the new service. Moreover, the intervention brought savings in travel time and costs for patients and their caregivers. These were computed as a cost for the control group. The monetary equivalent for the time spent by the patients and informal caregivers was calculated using the minimal interprofessional wages for the year 2016 and resulted in an hourly wage of €6.07 (approximately US $7.07).

All costs were homogenized per patient and per year. Bed days of each group were multiplied by the estimated cost per bed‐day in Spain (€733.56, approximately US $854.91).

### Analyses

Categorical variables were described as frequency and percentage of available data, whereas quantitative variables were described as mean and standard deviation or median and interquartile range; 95% confidence intervals were provided for mean differences. Between-group differences regarding the proportions of each category were compared using the chi-square test, whereas quantitative variables were compared using the *t* test, analysis of variance, or their nonparametric counterparts (Mann-Whitney *U* test and Kruskal-Wallis test, respectively). Normality was assessed using the Kolmogorov-Smirnov test [41]. In all comparisons, the significance threshold was set at a 2-sided α=.05. Descriptive and comparative analyses were performed using SPSS software (version 17.0; SPSS Inc).

Cost-effectiveness analysis was performed using Monitoring and Assessment Framework for the European Innovation Partnership on Active and Healthy Ageing (MAFEIP) [42]. MAFEIP is a free web-based tool promoted by the European Commission aimed at performing cost-utility analysis to estimate health outcomes and resource usage of a large sample of information and communication technology–enabled health and social care innovations, developed and implemented in the context of the European Innovation Partnership on Active and Healthy Ageing [42,43]. More precisely, the cost-effectiveness estimates are based on the principles of decision analytic modeling and a generic Markov model which provides the flexibility required to be tailored to the variety of solutions promoted by the European Innovation Partnership on Active and Healthy Ageing [44-46].

**Figure 1:**
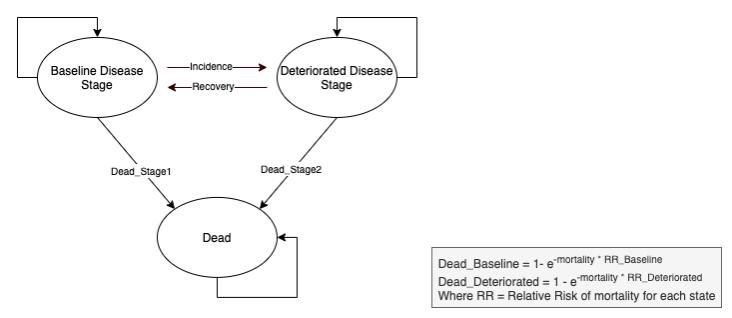
3-state Markov model applied for the BeyondSilos cost-effectiveness analysis.

Quality-adjusted life years were computed using change in the Barthel index as a proxy of utility as described by Kaambwa et al [47], and based on a 3-states Markov model: *baseline disease stage* (the patient remains in the same state or improves), *deteriorated disease stage* (the patient worsens), and *dead* (Figure 1). The 3 states led to the corresponding transition probabilities: *recovery* (improving or remaining the same state), *incidence* (worsening), and *death*. Mortality rates were internally calculated by the MAFEIP tool using all-cause mortality rates (age- and sex-dependent) extracted from the Human Mortality Database. Discount factors for health outcomes and costs were both set to 3% following recommendations from local Health Technology Assessment authorities. In order to estimate the incremental costs and outcomes associated with the intervention, we ran the model over a 40-year time horizon, following the proposed standardization for economic analysis of health technologies in Spain, which recommends assessing the costs and benefits on a time horizon that covers the entire lifespan of the patients affected [48-50].

## Results

### Participant Characteristics

**Figure 2:**
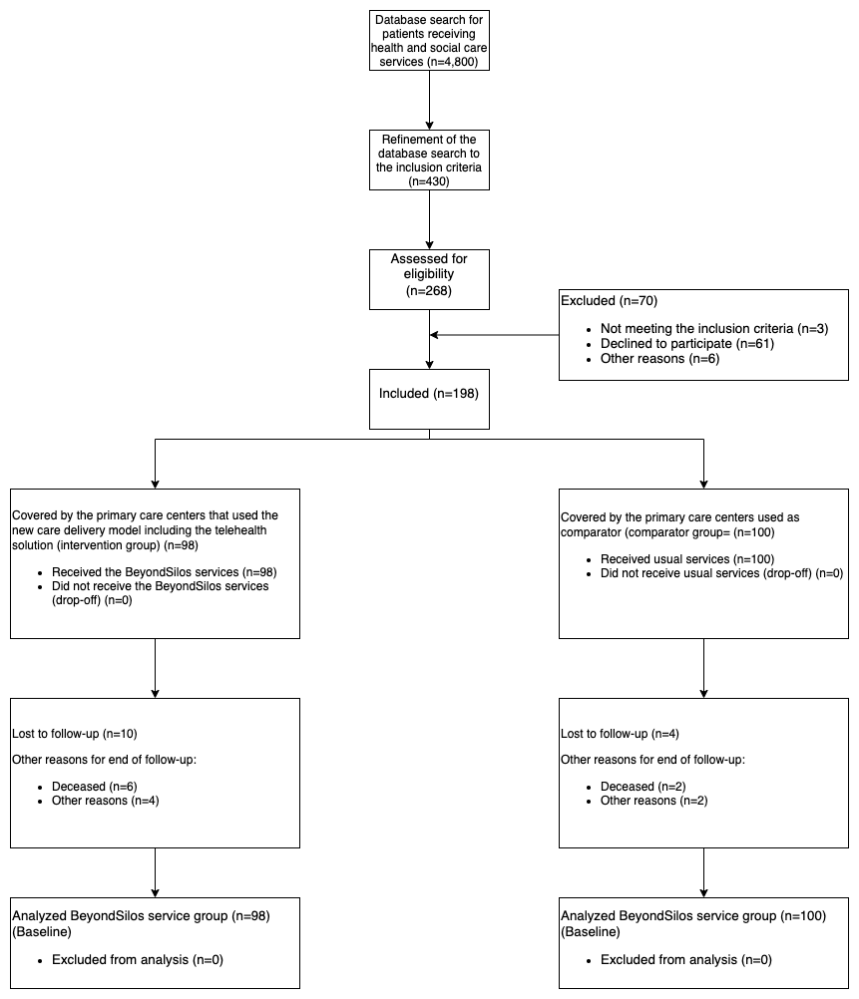
Flowchart of participant recruitment for the BeyondSilos project.

Of the 268 individuals considered for eligibility, 70 were excluded, resulting in a study sample of 198 patients: 98 (49.5%) were managed within the BeyondSilos project (intervention group) and 100 (50.5%) were managed according to usual care (comparator group) (Figure 2).

Table 1 summarizes the sociodemographic characteristics of study participants. Participants in the 2 study groups were balanced regarding education level and household income, but the intervention group tended to be overrepresented by older, female, and widowed individuals.

**Table 1:** Demographic baseline characteristics of the total sample (N=198).

Characteristics		Intervention group (n=98)	Comparator group (n=100)	*P* value
**Gender, n (%)**				.02
	Male	26 (26)	43 (43)	
	Female	72 (74)	57 (57)	
Age (years), median (IQR)		85.5 (7.3)	82.8 (8.3)	.01
**Age group (years), n (%)**				.003
	<65	1 (1.0)	0 (0)	
	65-75	7 (7.1)	24 (24.0)	
	>75	90 (91.8)	76 (76.0)	
**Marital status, n (%)**				.053
	Never married	2 (2.0)	3 (3.0)	
	Currently married	30 (30.6)	47 (47.0)	
	Separated	0	3 (3.0)	
	Divorced	2 (2.0)	1 (1.0)	
	Widowed	63 (64.3)	46 (46.0)	
	Cohabitating	1 (1.0)	0 (0)	
**Education level, n (%)**				.23
	Less than primary school	50 (53.2)	40 (40.8)	
	Primary school	30 (31.9)	42 (42.9)	
	Secondary school	5 (5.3)	10 (10.2)	
	High school	7 (7.4)	4 (4.1)	
	College/university	2 (2.1)	1 (1.0)	
	Post graduate degree	0 (0)	1 (1.0)	
**Household income (€^a^ yearly), median (IQR)**				.96
	0-6999	7 (13.2)	11 (14.7)	
	7000-13,999	32 (60.4)	45 (60.0)	
	14,000-19,999	12 (22.6)	15 (20.0)	
	20,000 or more	2 (3.8)	4 (5.3)	

^a^An approximate exchange rate of €1 to US $1.17 was applicable at the time of publication.

At baseline, study participants in both groups had a median of 3 comorbidities (IQR 2-4), with no significant differences regarding either the number of comorbidities (*P*=.96) or the prevalence of each comorbidity, except malignancies, which were 2.6-fold more frequent among those in the intervention group (Table 2). The mean Charlson Comorbidity index was 4.42 (SD 2.34) and 4.31 (SD 1.81) for the intervention and comparator groups, respectively (*P=*.79). Congestive heart failure was the most prevalent comorbidity in both study groups. The intervention group had significantly lower Barthel index scores (*P*=.001) and higher Geriatric Depression Scale scores (*P*=.002). This trend was not observed for the Instrumental Activities of Daily Living (*P*=.44).

**Table 2:** Clinical characteristics of study participants at baseline (N=198).

Characteristics		Intervention group (n=98)	Comparator group (n=100)	*P* value
**Comorbidities, n (%)**				
	Myocardial infarction	17 (17.3)	23 (23.0)	.32
	Congestive heart failure	61 (62.2)	71 (71.0)	.19
	Peripheral vascular disease	1 (1.0)	3 (3.0)	.33
	Cerebrovascular disease	43 (44.3)	25 (25.0)	.004
	Dementia	3 (3.1)	5 (5.0)	.49
	Chronic pulmonary disease	1 (1.0)	3 (3.0)	.33
	Rheumatic disease	3 (3.1)	10 (10.0)	.051
	Peptic ulcer disease	19 (19.6)	16 (16.0)	.51
	Mild liver disease	22 (22.7)	34 (34.0)	.08
	Diabetes without chronic complication	25 (26)	27 (27.0)	.88
	Diabetes with chronic complication	31 (32.0)	19 (19.0)	.04
	Hemiplegia or paraplegia	28 (28.9)	37 (37.0)	.22
	Renal disease	1 (1.0)	1 (1.0)	.99
	Malignancies^a^	23 (23.7)	9 (9.0)	.005
	Moderate or severe liver disease	3 (3.1)	4 (4.0)	.73
	Metastatic solid tumor	13 (13.4)	12 (12.0)	.77
**Anthropometric and laboratory exams, mean (SD)**				
	Body mass index (kg/m^2^)	28.8 (4.8)	27.3 (5.4)	.02
	Blood glucose (mg/dL)	110.8 (34.6)	116.9 (44.5)	.44
	HbA_1c_^b^ (%)	6.82 (1.70)	7.45 (1.81)	.11
	eGFR (mg/dL/1.73 m^2^)	75.9 (38.1)	74.4 (43.2)	.40
**Tobacco use, n (%)**				.12
	Never	75 (79.8)	69 (69.0)	
	Former	19 (20.2)	29 (29.0)	
	Current smoker	0 (0)	2 (2.0)	
	E-cigarette	0 (0)	0 (0)	
	Other	0 (0)	0 (0)	
**Alcohol drinking (weekly drinks past 12 months), n (%)**				.02
	None	87 (88.8)	80 (80.0)	
	<1	6 (6.1)	6 (6.0)	
	1-7	3 (3.1)	14 (14)	
	8-14	2 (2.0)	0 (0)	
	15-21	0 (0)	0 (0)	
	>21	0 (0)	0 (0)	
**Assessment scores, mean (SD)**				
	Barthel index	44.66 (27.37)	71.58 (27.95)	.001
	Instrumental Activities of Daily Living	1.45 (1.74)	2.94 (2.55)	.44
	Geriatric Depression Scale	7.23 (3.47)	6.11 (3.51)	.002

^a^Any malignancy, including lymphoma and leukemia, except malignant neoplasm of skin.

^b^HbA_1c_: glycohemoglobin.

^c^eGFR: estimated glomerular filtration rate.

### Clinical Effectiveness

The Barthel index remained unchanged throughout the follow-up period in the intervention group (mean change from enrolment to end was 0.14, 95% CI –4.51 to 4.78; *P=*.95), but decreased in the comparator group (mean change –3.23, 95% CI –5.34 to –1.11; *P=*.003). The score of the Instrumental Activities of Daily Living significantly decreased in both groups: mean change of –0.23 (95% CI –0.44 to –0.02) in the intervention group (*P=*.03) and –0.33 (95% CI –0.46 to –0.20) in the comparator group (*P<*.001). The Geriatric Depression Scale score did not significantly change in either the intervention group (mean change 0.28, 95% CI –0.44 to 1.01; *P=*.44) or the comparator group (mean change –0.29, 95% CI –0.59 to 0.01; *P=*.06). None of the deaths were deemed to be related to the intervention or likely to been preventable with the intervention.

### Resource Utilization

During the 8 months of follow-up, the study participants contacted the health care or social professionals 5209 times: 2556 times in the intervention group and 2653 times in the comparator group. The contact profile of the 2 groups differed significantly regarding the type of professional, the planned/unplanned contact, and the setting of contacts (Table 3). Overall, participants in the intervention group tended to have contact more with their general practitioner and the social worker, and less with the specialists. Regarding the type of visit, participants in the intervention group tended to have more planned visits, predominantly at home, compared to those of the comparator group.

**Table 3:** Resource utilization of study participants (N=198).

Resource use			Intervention group (n=98)	Comparator group (n=100)	*P* value
**Hospitalization**					
	Hospitalized patients, n (%)		32 (32.7)	45 (45.0)	.08
	Length of hospital stay per admission (days), mean (SD)		5.84 (8.81)	2.3 (2.8)	.02
	Length of hospital stay per patient (days), mean (SD)		12.9 (15.0)	6.36 (9.0)	.02
	Time to first admission (days), mean (SD)		56.3 (57.9)	70.8 (59.3)	.31
	Admissions per patient (all patients), mean (SD)		0.85 (1.61)	1.12 (2.10)	.17
	Readmissions within 30 days per patient, mean (SD)		1.73 (1.78)	2.11 (2.74)	.96
	**Type of admission, n (%)**				.63
		Planned	24 (28.9)	36 (32.1)	
		Unplanned	59 (71.1)	76 (67.9)	
	Annual length of hospital stay (unplanned admissions), mean (SD)		1.58 (5.15)	0.65 (1.41)	.74
**Interaction with health and social professional**					
	**Type of professional, n (%)**				
		General practitioners	895 (34.2)	670 (23.3)	<.001
		Specialists	116 (4.4)	225 (7.8)	<.001
		Nurses	1504 (57.5)	1901 (66.1)	<.001
		Other health care provider	25 (1.0)	39 (1.4)	.17
		Social workers	76 (2.9)	42 (1.5)	<.001
		Volunteers	N/A^a^	N/A	N/A
	**Type of anticipation, n (%)**				<.001
		Planned	1677 (93.2)	1359 (87.6)	
		Unplanned	123 (6.8)	193 (12.4)	
	**Setting of contacts, n (%)**				
		Physical meeting out of home	239 (9.4)	563 (21.2)	<.001
		Home visit	1089 (42.6)	687 (25.9)	<.001
		Telephone	759 (29.7)	535 (20.2)	<.001
		Writing (email, SMS text message, etc)	463 (18.1)	857 (32.3)	<.001
		Other	6 (0.2)	8 (0.3)	.82
	**Annual rates for contacts, mean (SD)**				
		Annual contacts rate	51.0 (36.1)	53.1 (40.3)	.85
		Annual unplanned contacts rate	2.4 (3.5)	3.8 (5.3)	.07
		Annual physical contacts rate	24.9 (23.5)	23.4 (18.1)	.66

^a^N/A: not applicable.

### Cost-Effectiveness Analysis

Table 4 summarizes the costs with transition probabilities between the 3 health states of the Markov model used as inputs for the MAFEIP tool. Although the expenditures shared by the 2 care models were very similar, the intervention group was associated with an extra cost, resulting in an incremental cost of €4755 (approximately US $5542). The increase of costs was associated to the extra home visits and general practitioner contacts associated to the training and usage of the telehealth technology (for a detailed table of costs see Multimedia Appendix 3).

**Table 4:** Input used for the cost-effectiveness analysis based on the 3-state Markov model (N=198).

Input			Intervention group (n=98)		Comparator group (n=100)
**Transition probabilities, %**					
	Incidence	34		36	
	Recovery	66		64	
**Relative risk (mortality)**					
	Baseline disease stage	1.005		1.005	
	Deteriorated disease stage	1.005		1.005	
**Utility after intervention**					
	Baseline disease stage	0.56		0.45	
	Deteriorated disease stage	0.3		0.33	
**Costs, € ($)^a^**					
	One-off cost per patient (intervention)	1268.89 (1484.60)		N/A^b^	
	Recurring cost per patient/year (intervention)	230.40 (269.57)		N/A	
	Health care cost—baseline disease stage	5664.89 (6627.92)		5198.62 (6082.39)	
	Health care cost—deteriorated disease stage	4502.89 (5268.38)		5221.69 (6109.38)	
	Societal cost—baseline disease stage	5953.15 (6965.19)		5259.14 (6153.19)	
	Societal cost—deteriorated disease stage	4791.15 (5605.65)		5282.21 (6180.19)	

^a^An approximate exchange rate of €1 to US $1.17 was applicable at the time of publication.

^b^N/A: not applicable.

**Figure 3:**
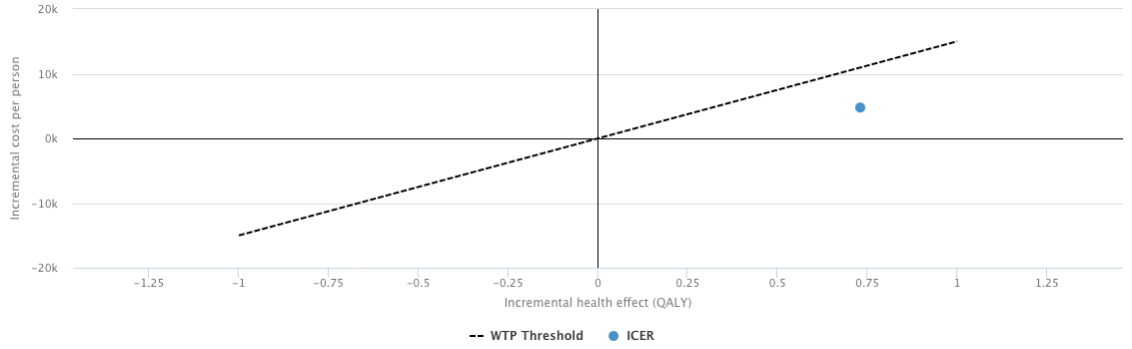
Cost-effectiveness plane for a willingness-to-pay of €15,000 (approximately US $17,481)/quality-adjusted life year. ICER: incremental cost-effectiveness ratio; QALY: quality-adjusted life year; WTP: willingness-to-pay.

The effectiveness, computed based on transition probabilities between the 3 states of the Markov model, was also higher in the intervention group, yielding an incremental effect of 0.731. Overall, the incremental cost-effectiveness ratio was €6505.52 (approximately US $7582), making the intervention more effective than usual care for all willingness-to-pay thresholds above €6500 (approximately US $7575) per quality-adjusted life year (Figure 3).

**Figure 4:**
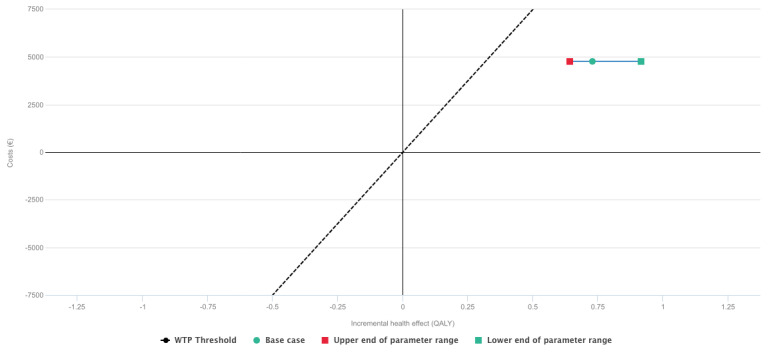
Sensitivity analysis showing effects between 0% and 5% change in utilities—willingness-to-pay of €15,000 (approximately US $17,481)/quality-adjusted life year. QALY: quality-adjusted life year; WTP: willingness-to-pay.

The sensitivity analysis showed that a change between 0% and 5% in the utility in the baseline health for the intervention group would place the incremental cost-effectiveness ratio still below the willingness-to-pay threshold of €15,000 (approximately US $17,481)/quality-adjusted life year (Figure 4).

**Figure 5:**
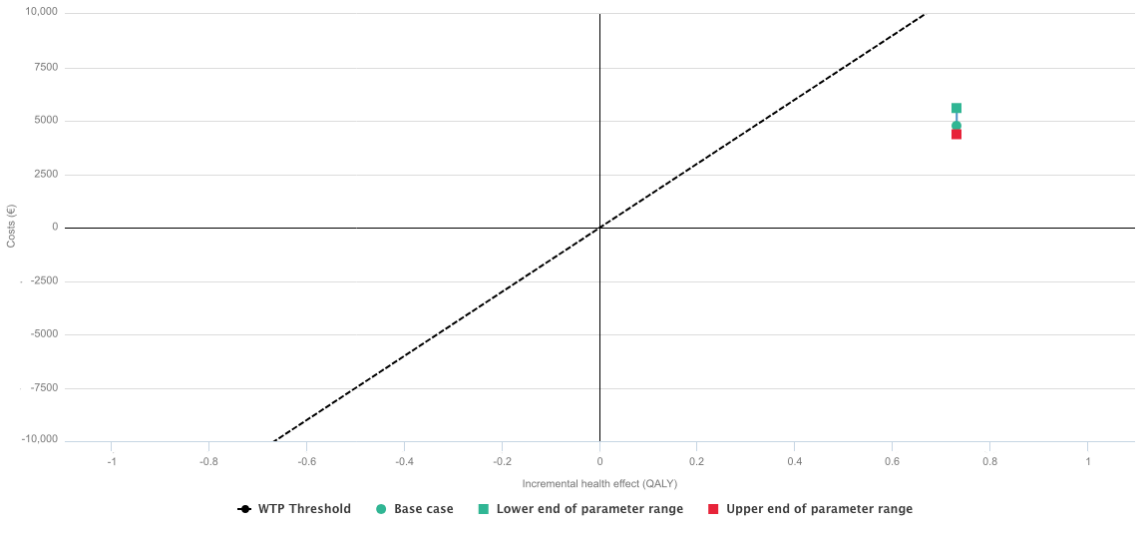
Sensitivity analysis showing effects between 0% and 5% change in costs—willingness-to-pay of €15,000 (approximately US $17,481)/quality-adjusted life year. QALY: quality-adjusted life year; WTP: willingness-to-pay.

Similarly, a change between 0% and 5% in the health care costs would not affect the result (Figure 5).

## Discussion

### Summary of Main Results

In this observational prospective cohort study, we found that the addition of an information and communication technology solution (which also involved the third sector) to a basic integrated care model was more effective than integration only in terms of transition between health states established with the Barthel index and the Instrumental Activities of Daily Living. The superiority of the BeyondSilos intervention was confirmed by all willingness-to-pay thresholds above €6500 (approximately US $7575) per quality-adjusted life year, far below the €30,000 (approximately US $34,963) threshold traditionally considered in Spain [51].

Besides the specific context of the pilot site, these results must be interpreted by bearing in mind the challenges of assessing cost-effectiveness of a complex intervention (such as an integrated care model). First, the complexity of both the intervention and the usual care model (in this case, an integrated care framework) often blurs the different contribution of each element to costs [7,15]. This also applies to stakeholders in the third sector (volunteer care), which cannot be easily quantified. Furthermore, it is worth mentioning that quality-adjusted life years may not always be a useful indicator for decision making at the level of provider organizations, particularly when (1) the delivery of care is already constrained by decisions at national or regional level [50] and (2) additional factors such as patient and provider satisfaction need to be taken into account.

### Contextualization of the Badalona Pilot Within the BeyondSilos Project

An important characteristic of projects aimed at implementing integrated care strategies is the need of tailor the overarching plan and methodology to the organizational framework of each area. Therefore, considering the expected differences between pilot sites in this regard, the original purpose was to provide a general integrated care framework so that pilot sites could tailor it to their health care environment. The most remarkable characteristic of Badalona pilot site was that, unlike other pilot sites enrolled in the BeyondSilos project, it was already delivering both health and social care services based on an integrated care approach. In this context, the BeyondSilos project added only 2 remarkable improvements compared with usual care: (1) a deeper commitment of the third-sector organizations and (2) the use of information and communication technology to enhance domiciliary care. The fact that the pilot site already operated under an integrated care approach had the advantage that the health care team was already used to integrated pathways, thus facilitating the incorporation of additional integrated care elements into the organizational model. However, this feature brought the trial to a challenging scenario in which the comparator (ie, comparator group) already included social care within the integrated care approach, thus reducing the benefits of the BeyondSilos model.

### Strengths, Limitations, and Future Work

Our analysis was strengthened by the appropriate balance between the primary care centers that piloted the information and communication technology–based intervention and those acting as comparators (paired by socioeconomic status). Although this approach did not preclude baseline differences in some demographic and clinical characteristics, the study groups were balanced regarding sociodemographic characteristics that may influence attitudes toward information and communication technology, such as income and education level.

On the other hand, studies investigating the effectiveness of integrated care models have to deal with the difficulty of establishing an adequate comparator [7]. As a rule of thumb, usual care is the recommended comparator, but this had different meanings for the various pilot sites in the BeyondSilos project, with some comparing nonintegrated and integrated care models, and others―as in our pilot site―comparing 2 integrated care models with different intensities. The last approach has been increasingly used as more areas adopt integrated care approaches [52,53], although there is less room for improvement. Another challenge of the assessment of integrated care models includes patient profiles, often characterized by a multimorbid conditions, which may be rather heterogeneous [7,15]. In our study, the baseline demographic and clinical characteristics of patients in the 2 groups were similar, but patients in the intervention group tended to be female, older, widowed, more dependent, and with higher depression scores. These differences, likely because of the real-life setting, should be carefully considered when appraising the scope of our results. Specifically, the characteristics of the intervention group might be associated with a higher need of formal care and information and communication technology solutions than that in the control group, thus potentially shading the actual benefits of the intervention.

Keeping these limitations in mind, we found that the frequency of planned and home visit contacts was significantly higher in the intervention group (*P*<.001). Although this trend might be influenced by the higher complexity of patients in the intervention group, health care professionals explicitly explained that the usage of information and communication technology required more of their time and they were afraid that information and communication technology may replace their jobs. This attitude, together with the usual resistance of care recipients to losing contact with their formal caregivers [54,55], was likely to hinder the reduction of home visits that is expected with telemonitoring. Of note, the lack of differences in the estimated annual rates suggests that this phenomenon was not homogeneous throughout the follow-up period, being more pervasive during the first stages of the intervention. The temporal patterns of this attitude may reflect a certain resistance of professional staff to trust the new information and communication technology–supported integrated care model (ie, not fully taking advantage of the telemonitoring solution thus not abandoning the routine cadence of home care visits). Besides being a lesson for future implementation of information and communication technology solutions, this observation suggests that, in our study, uncontrolled factors such as the personal commitment of professionals to the project might influence the apparent cost-effectiveness of an information and communication technology solution, potentially overriding other factors such as patient characteristics. Future evaluations based on multicriteria decision analyses may provide interesting insights regarding the implementation of information and communication technology–enhanced integrated care programs [56].

### Conclusion

Our study provided evidence regarding the clinical effectiveness and cost-effectiveness of an information and communication technology–enhanced integrated care model that enables telemonitoring and increases the intensity of integrated care by involving organizations of the third sector in the management of older patients in a domiciliary care setting. The cost-effectiveness analysis placed the intervention as more effective than usual care―and reasonably inexpensive. However, our findings confirm the difficulties of assessing the effectiveness of interventions and suggest that the beneficial effects of a new care model strongly depend on the commitment of health and social care professionals with the model.

## Appendix

Multimedia Appendix 1 BeyondSilos integrated common care pathways for home care support.

Multimedia Appendix 2 Telehealth solution used within the BeyondSilos project (Badalona pilot site).

Multimedia Appendix 3 Tables of costs.

## Abbreviations

BSA: Badalona Serveis Assistencials
MAFEIP: Monitoring and Assessment Framework for the European Innovation Partnership on Active and Healthy Ageing
