# Supplementary material for: BeyondSilos, a Telehealth-Enhanced Integrated Care Model in the Domiciliary Setting for Older Patients: Observational Prospective Cohort Study for Effectiveness and Cost-Effectiveness Assessments
Source: JMIR Med Inform. 2020 Oct 6;8(10):e20938. doi: 10.2196/20938 (PMC7576466; doi:10.2196/20938)
Supplement: Multimedia Appendix 1 [file medinform_v8i10e20938_app1.pdf]

1. Integrated common care pathways for home care support

It should be noted that the concept of 'care pathways' has been used differently in different contexts, both in health care and in social care (where in several countries this concept may not be used at all). For the purposes of BeyondSilos, it is intended to be used in a pragmatic manner. It is considered as an enabler for a systematic description of a defined sequence of actions carried out by collaborating parties. In the following descriptions, generic terminology is used whenever possible in order to avoid a terminological bias towards the health or social care domains.

Integrated Short-term Home Care Support (Pathway #1 - ICP short)

This pathway is designed to support people who have experienced a significant 'event' such as stroke, MI, fractured neck of femur, or other injuries and illnesses which impact adversely on the person's ability to live independently. The activities in the pathway focus on delivering time-limited interventions, services, care and support such as the following:

- Rehabilitation: stroke, MI, fractured neck of femur (after hospital stay, ER).
- Reablement: other injuries and illnesses.
- Structured patient education programmes: pulmonary rehabilitation, diabetes education programme.

Following completion of the time-limited care provision (or at any time point within the period), the individual will be reviewed and either:

- discharged back to 'usual' care.
- referred onto the Long-Term Care Pathway.

Integrated Long-term Home Care Support (Pathway #2 - ICP LTCare)

This pathway is designed to support people living with complex needs whose joint care assessment indicates that ongoing health and social care services and wellbeing assistance is required (delivered and/or funded by public sector and/or third sector organisations). As and when the individual experiences an 'event' which impacts adversely on their health and wellbeing (a likely occurrence for the BeyondSilos population cohort), the interventions, services, care and support to maintain the person living in their own home will be reviewed and adjusted temporarily in one or more of the following ways:

- The person's care practitioners or key worker / case manager / care coordinator may make more frequent checks on the person's health and wellbeing (face-to-face visits, telephone or virtual).
- The person may receive additional interventions, drugs, services, care or support.

Once the individual has been assessed as having recovered from the 'event', their care plan will once again be reviewed, and any necessary adjustments to their ongoing interventions, services, care and support made. If the individual has a severe exacerbation they may, of course be admitted to hospital.

If the person experiences a significant 'event' such as a stroke, MI, or fractured neck of femur, they should be considered to have transitioned onto the Short Term Care Pathway, where all their long term complex needs will be managed in addition to any additional needs following the occurrence of the significant event.

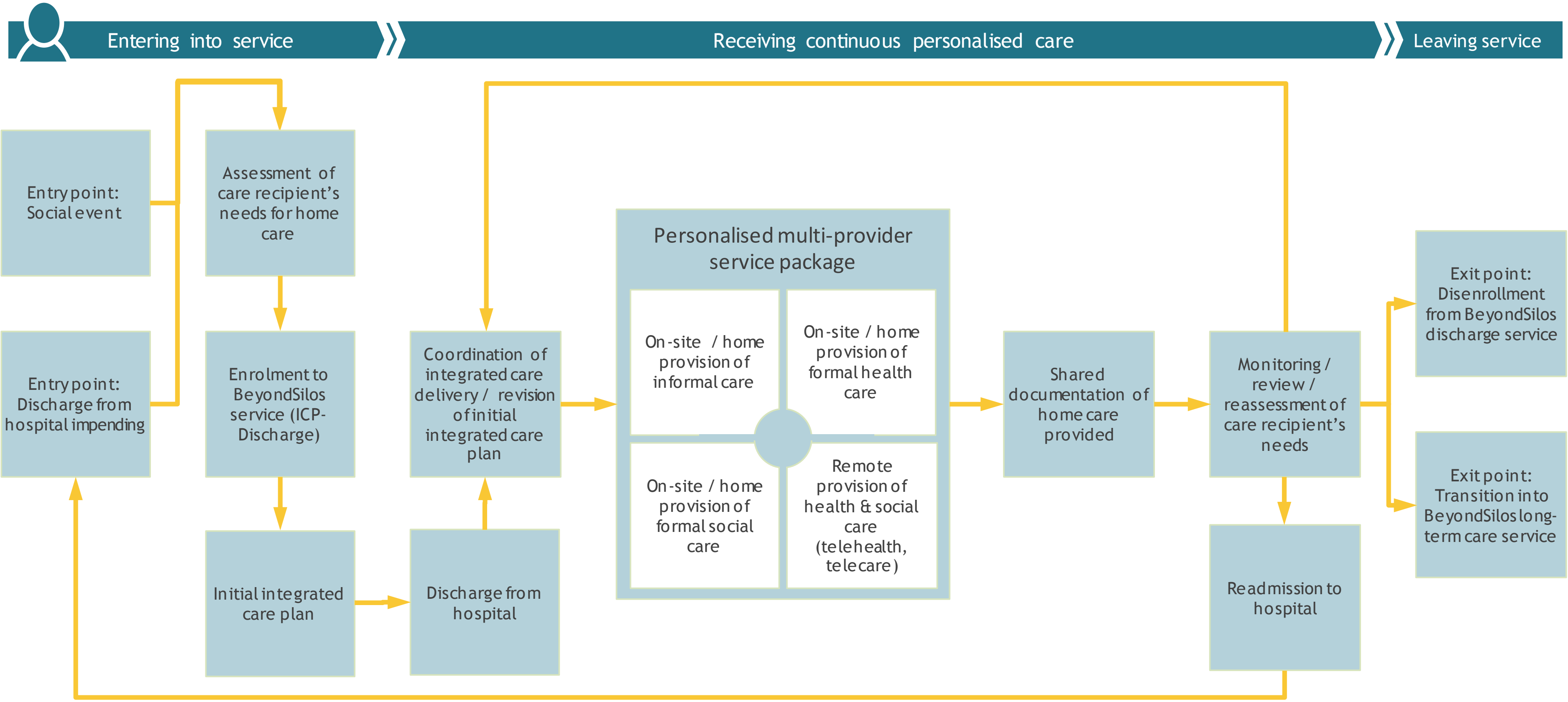

Figure 1: Integrated Short-term Home Care Support (Pathway #1 - ICP short)

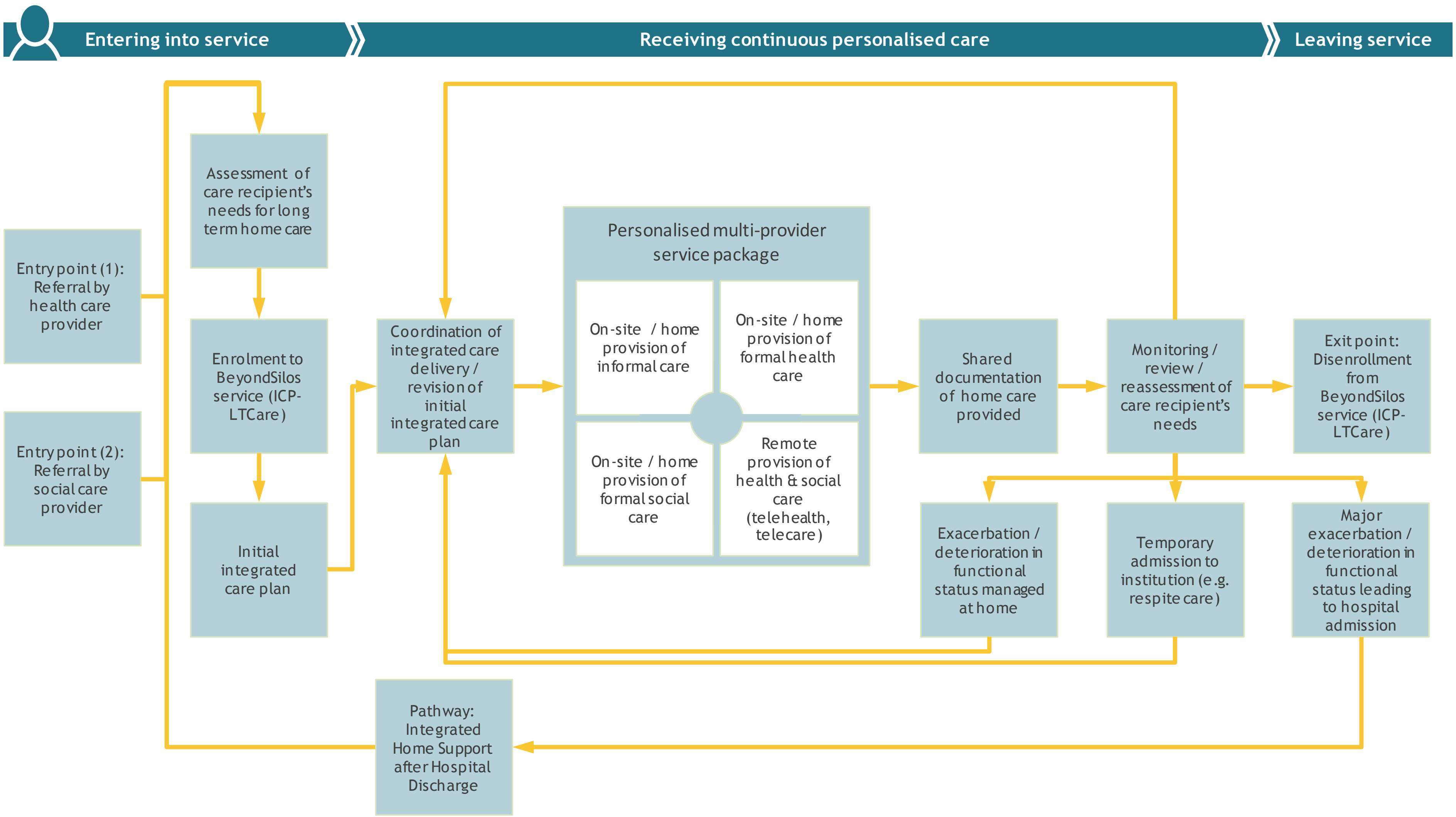

Figure 2: Integrated Long-term Home Care Support (Pathway #2 - ICP LTCare)

1.1 Pathway elements

1.1.1 Entry point

Entry points into both BeyondSilos pathways may vary according to individual service users and pilot regions. Individual end users may for instance be referred to the BeyondSilos service by health or social care professionals already working with them in other contexts. Depending on the “business” model intended to be adopted for mainstreaming purposes, direct subscription to the BeyondSilos services by older people and/or their family may be an option as well. Examples of the latter can, for instance, be found in countries where non-medical telecare schemes (e.g. social alarms, home security sensing) are usually not provided as a public duty under the auspices of the municipality or regional government.

When it comes to hospital discharge in particular, the entry point is usually defined by an impending discharge event. Here, the BeyondSilos pathway would need to link in an appropriate manner into discharge pathways already existing internally to a given hospital.

1.1.2 Assessment of the service user’s needs for integrated home care

This step focuses on assessing the individual service user in relation to any home care needs they may have. This will usually be a systematic process which relies on pre-defined assessment criteria / procedures. These enable identification of health-related needs as well as needs for other forms of home support. Implementation of this process is thus likely to require involvement of multi-disciplinary expertise. Generally, it should focus on client-specific risk factors and service outcomes that can be realistically anticipated from relevant professional perspectives for the individual service user.

1.1.3 Enrolment into BeyondSilos services

This element stands for the process by which individuals register to become a participant in the service to be piloted. Appropriate eligibility criteria, consent procedures, etc., need to be available and applied.

1.1.4 Initial integrated home care plan

This step focuses on an initial plan for joined-up provision of home support through the BeyondSilos service. It responds to the previously identified care needs in a holistic and integrated manner. The documentation of the plan is an analytical process of activity designed to establish a course of client care, potentially establishing priorities and selecting a course of action from identified alternatives. The results are documented in a systematic manner and set out inputs, delivery, management and organisation of service delivery to the home.

1.1.5 Discharge from hospital (ICP-Short only)

In the case of the generic pathway #1 (ICP Short), the coordinated transition of the patient from hospital or ER to home is supposed to be critical to his/her health and well-being. Patients, family caregivers and professional care providers all play roles in maintaining a patient's health after

discharge. Coordinated discharge planning is seen as a significant step towards an integrated overall care plan.

1.1.6 Ongoing coordination of integrated care delivery / revision of the initial care plan

This element focuses on ongoing tracking of BeyondSilos users when they receive professional home care and/or informal support from different parties as identified in the initial care plan. It enables professional and informal carers to coordinate delivery of required care interventions, and to utilise all potentially available resources. The main aim is to effectively manage a system of targeted collaboration over time, thereby involving all relevant parties including the BeyondSilos service users themselves. A "link man" function (sometimes referred to as a case manager) may need to be established to ensure that any changing needs of the BeyondSilos users are identified. In response, the right mix of medical, social and informal care in line with user expectations is delivered. Beyond the involvement of health and social care expertise, a clear assignment of responsibilities is required when it comes to decision making on any care plan adaptations potentially required.

1.1.7 On-site provision of formal healthcare and social care

This step focuses on coordinated performance of care-related measures through professional health and social care staff and informal carers in the older person's home. The range of tasks may require both medical interventions and/or non-medical custodial tasks and/or non-skilled care, such as assisting with activities of daily living such as dressing, bathing, and using the bathroom.

1.1.8 On-site provision of informal care

Beyond care provided by professional care staff, non-professional care may be provided by family members and/or other informal carers. This may include medical care tasks (e.g. taking vital sign measurements) or non-medical custodial tasks.

1.1.9 Remote provision of care to the home

The remote exchange of data and/or electronic communication between the BeyondSilos service user and healthcare professionals is one example of remote provision of care. This may be necessary to assist in the diagnosis and/or management of a healthcare condition. Examples include blood pressure monitoring, blood glucose monitoring, and medication reminders. Potentially, remote transmission of patient information, e.g. symptom reports, to a clinician for expert diagnosis and/or management may be involved as well.

On the other hand, remote care provision may include ICT-based services involving data exchange and/or electronic communications between the BeyondSilos service user and non-medical professionals (telecare). Here, examples include (active) push-button alarms and automatic (passive) monitoring of changes in an individual's condition or lifestyle, including emergencies, to manage the risks of independent living. The latter may require installation of one or more types of sensors in the service recipient's home, such as movement sensors, falls sensors, bed/chair occupancy sensors and the like.

1.1.10 Integrated documentation of provided home care

The documentation of any care-related measures performed for the patient needs to be available in an integrated manner. It serves as a basis for ongoing decision-making within the overall care process between all involved carers.

A number of aspects may deserve attention, such as the tailored presentation of information for the needs of healthcare professionals, social care professionals or informal carers. This may take the form of a client / patient summary. The eligibility for reimbursement of certain care acts is another example. Documentation can also serve auditing purposes when it comes to the quality of care provided.

In addition to care interventions, documentation may also include information relating to various types of assessments performed at the point of care, e.g. fall risk assessment, periodic psychoactive summary, restraint needs assessment, pain assessment for those with communication barriers and the like.

1.1.11 Control / reassessment

This step focuses on systematically monitoring documented care interventions and related outcomes, with a view to enabling meaningful adaptation of the initial care plan over time.

1.1.12 Temporary admission or re-admission to an institutional setting (ICT-LTCare only)

Depending on the BeyondSilos service user's status, a temporary admission or re-admission into a stationary care setting may be required, e.g. a day care centre or respite care. After the CR has been discharged from the institutional setting, their social and healthcare needs will be reassessed, and service delivery adapted accordingly.

1.1.13 Exacerbation / deterioration in functional status managed at home (ICT-LTCare only)

When it comes to exacerbations of the chronic condition(s), two different scenarios are distinguished: On the one hand, a minor deterioration in the health status or well-being of the patient will lead to the reassessment of their social and healthcare needs, and changes in services provided accordingly. The main aim, however, is to keep the CR in their own home for as long as possible, as this usually has a positive influence on the quality of life for the CR.

1.1.14 Exacerbation / deterioration in functional status leading to hospital admission (ICT-LTCare only)

On the other hand, exacerbations of the chronic condition(s) may lead to a temporary admission to a hospital in order to adequately help and support the CR. In this scenario, the CR will be transferred to the ICP-Short pathway.

1.1.15 Exit point

Exist points from the pathways may vary according to individual service users. When it comes to the acute pathway (ICP-Short) in particular, transition into the long-term home care pathway (ICP-LTCare) may happen at a certain point in time
