# Supplementary material for: BeyondSilos, a Telehealth-Enhanced Integrated Care Model in the Domiciliary Setting for Older Patients: Observational Prospective Cohort Study for Effectiveness and Cost-Effectiveness Assessments
Source: JMIR Med Inform. 2020 Oct 6;8(10):e20938. doi: 10.2196/20938 (PMC7576466; doi:10.2196/20938)
Supplement: Multimedia Appendix 2 [file medinform_v8i10e20938_app2.pdf]

1. Telehealth solution used within the BeyondSilos project (Badalona pilot site)

1.1 Health Insight Solutions Platform (HIS Portal)

This software supplies the medical and social service organisations with the necessary monitoring data, analytical tools, and communication procedures that enable the efficient planning, coordination and delivery of their services in the home environment.

All solutions can be integrated into the overall system and can be managed and controlled from the role-model based HIS Portal.

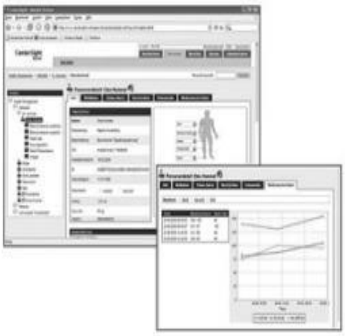

The web-based HIS Portal is a tool that supports medical practitioners, care providers, administrators and technical staff. It receives the measurement data from the different HIS Gateways. The patients' data is stored for further consultation by authorised users and can equally be used for analysis.

Notification threshold, trend alarms and communication channels can be adjusted individually for each citizen or each group of citizens. This feature allows preventative medical provision at the very moment that the monitored individual's state of health requires it. Individualised measurement schedules can also be set.

Automatic pdf reports can be sent to a predefined email or fax on a daily, weekly or monthly basis, e.g. to the doctor, to give a summarised overview of the measurement data.

The HIS Portal can also be used to enter notes that are visible to the other carers, or to attach documents, e.g. X-rays.

Multiple definable user and access authorisations safeguard sensitive data. It is possible to interface the HIS Portal to other systems such as Electronic Health Records.

The HIS Portal provides the following features:

- Administration management: management of users, patients, mobile devices, etc.
- Patient management: management of patient information, medical data, etc.
- Role model management: set up of access rights.
- Warning management: set up of warnings, conditions, transmissions, notifications etc.
- Monitoring data management: overview of data received by the HIS Portal.
- Report management: creation of reports with medical data or environmental data from patients.
- Configuration, set-up and device management: configuration mobile solution etc.

- Application management: reminder settings, videoconference, questionnaires etc.

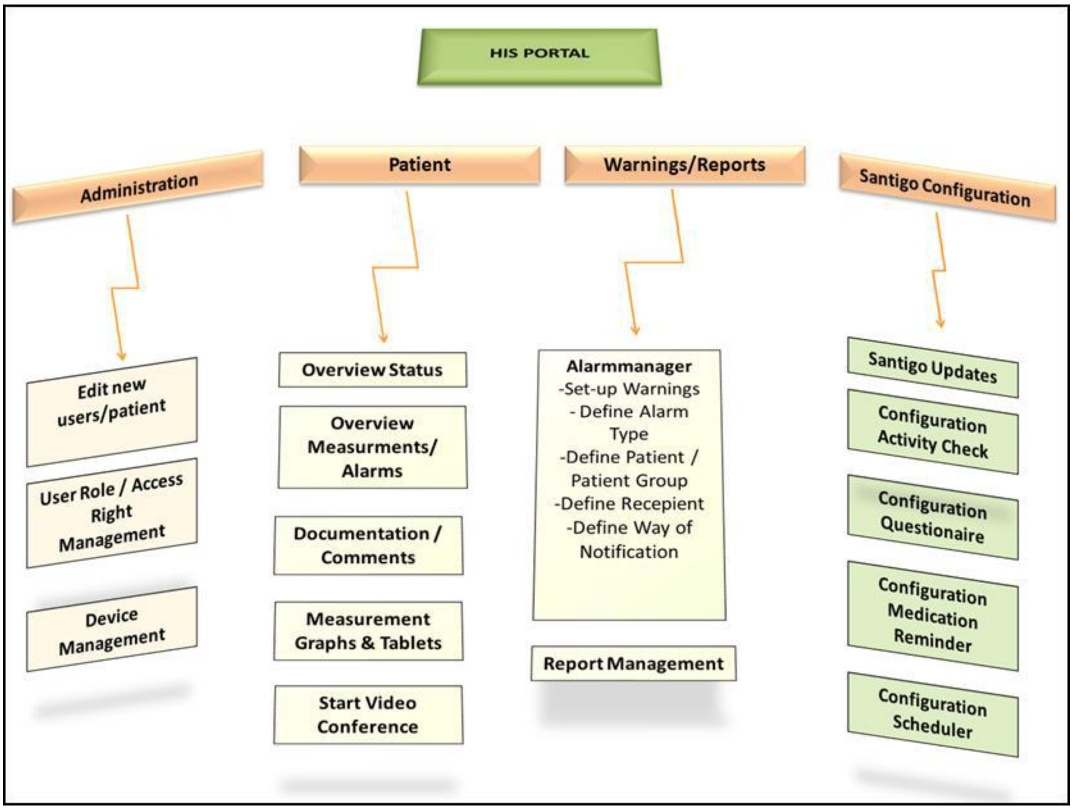

Figure 1: HIS Portal: functionalities overview

The most interesting features to define in more depth are the following:

- **Administration Management**

  "Administration Management" is an important tool in the organisation of the HIS Portal and the connected tablets / mobiles and vital monitors.

  - An overview of all patients, users and tablets / mobiles;
  - An overview of the existing user roles;
  - An overview of the registered users;
  - An overview of all patients;
  - An overview of all connected gateways;
  - System information;
  - Mobile Option configuration tool.

The main features of the administration section are the possibility to:

- Add / edit / delete patients;
- Add / edit / delete users / administrators, their access and their passwords;
- Add / change / delete users' rights and role models;
- Assign gateways to users;
- Add / delete folders and patient links.

- **Patient management**

The patient management section of the HIS Portal is designed to organise information about the patient; it contains all measurement data.

An overview chart in the patient sections displays the latest measurements with a time stamp, and the connected vital monitors. It also shows if the last measurements were in range or if they triggered an alarm. All active alarms are displayed as well.

The HIS Portal offers different administration tools which support and help to structure, administer and manage the Santigo Solution, patients, devices and incoming data.

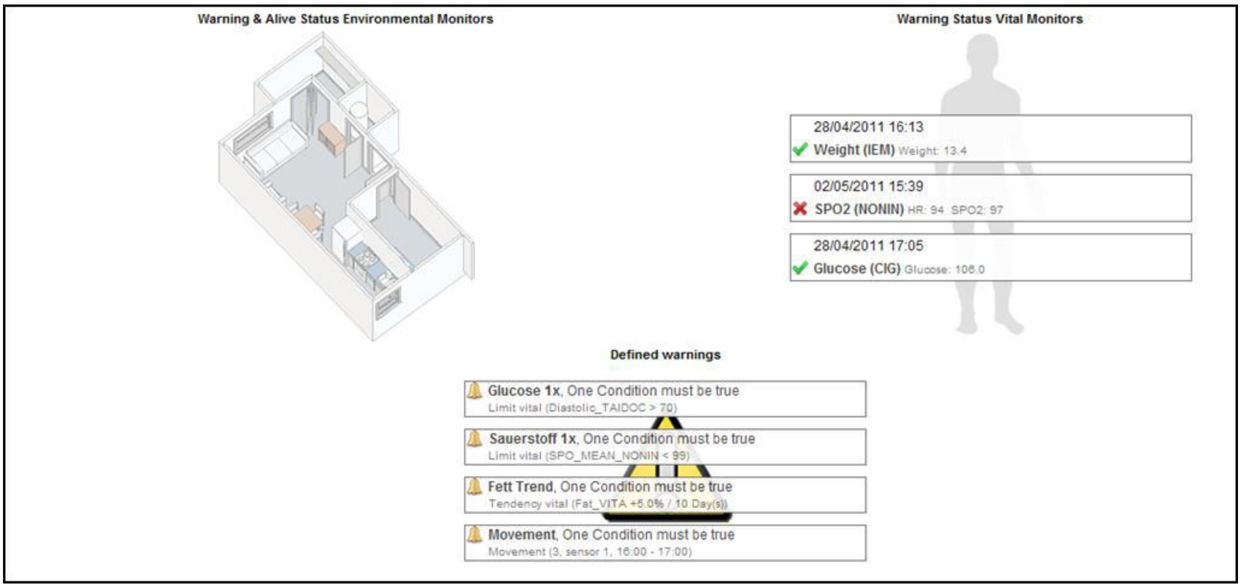

Figure 2: HIS Portal: alarms overview

- **Behavioural analysis**

The innovative Behavioural Analysis of the HIS Portal is based on different data which is collected by the different sensors. This could be a powerful tool help to minimise risk and optimise the service an elderly person needs.

The in-house observation of movements is performed by collecting motion events using motion detectors in different rooms of the living environment. Every motion detector sends detected motion to the HIS HomeBox. The HIS HomeBox collects the single events and sends them to the portal on an hourly basis. In the portal, a rules engine compares the movement pattern. If there is an irregularity out of the predefined range, a notification is generated, and an alarm is triggered.

In a first step, the software will identify and learn the standard motion pattern of movement in the home. This "learning step" will be done for every person individually, to identify the pattern. In the so-called "recall phase", the identification of a specific pattern is done automatically. For a better granularity in defining rules to trigger alert messages, it is possible to set parameters that control the sensitivity of the detection.

The movement pattern can be also combined with other data which is collected by the monitoring system. For example, vital measurements done in a predefined timeframe, reaction times of cognitive training games, results of door open/close monitors, etc. Every single parameter will be added to the analysis and will help to refine the prediction.

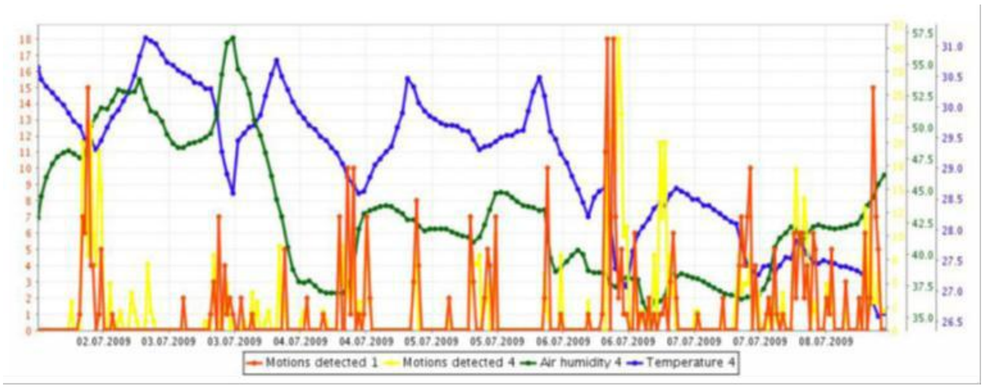

Figure 3: HIS Portal: behavioural analysis

The general overview of the architecture of the HIS Portal is shown in Figure 4 below.

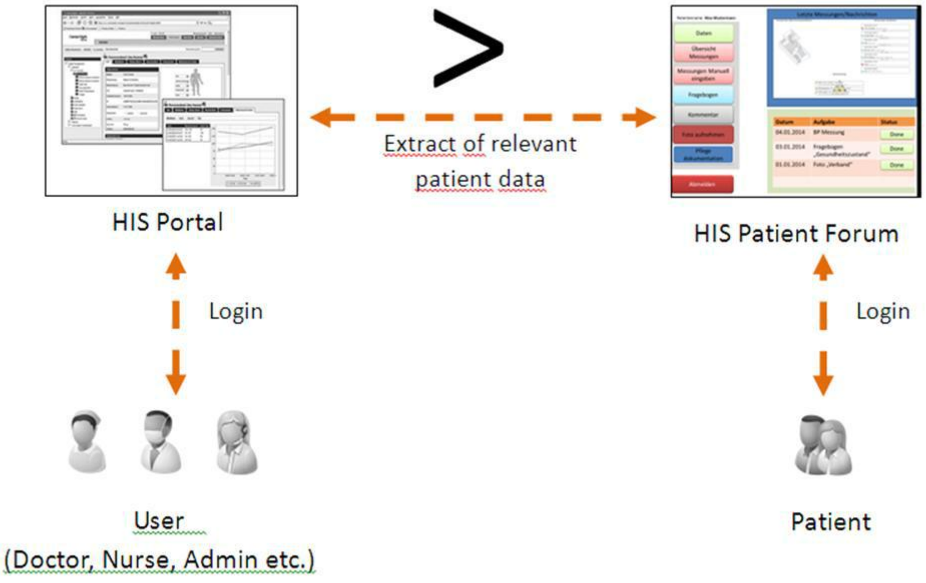

Figure 4: HIS Portal: general architecture

1.2 Health Insight Solution Santigo (HIS Santigo)

Up to this point, only the back-office of the solution has been shown; this is the one used by professionals. For patients, the proposed solution to interact with the HIS Portal is HIS Santigo.

With the HIS Santigo application, Health Insight Solutions provides a telehealth solution based on Android technology which can be run on Android based tablets or mobile phones.

It was developed specifically for citizens with chronic diseases, older people, and patients in rehabilitation.

In combination with the HIS Portal, the Santigo application provides the following care function modules, which can be combined depending on the requirements of the care process:

- Vital Measurements.
- Measurement History.
- Videoconference (optional).
- Medication reminder (optional).
- Activity check (optional).
- Questionnaires (optional).

HIS will provide a downloadable application, which can easily be installed on the tablets which fulfil the technical minimum requirements (e.g. Android 2.2 or higher, and Bluetooth 2.0 and higher). All tablets / mobiles will be set-up automatically in the HIS Portal and can then easily be linked to a patient.

The user-friendly administration of the solution, the easy to handle management of patients and devices, and the scalability as far as roll out is concerned, allows a successful integration into the health infrastructure.

Finally, the HIS Santigo solution and the HIS Portal allow to combine the single patient solution with a multi patient solution (Santigo Care) which was especially developed for caregivers. This flexibility enables institutions to meet their requirements regarding different roles of caregivers and citizens within a project, as shown below.

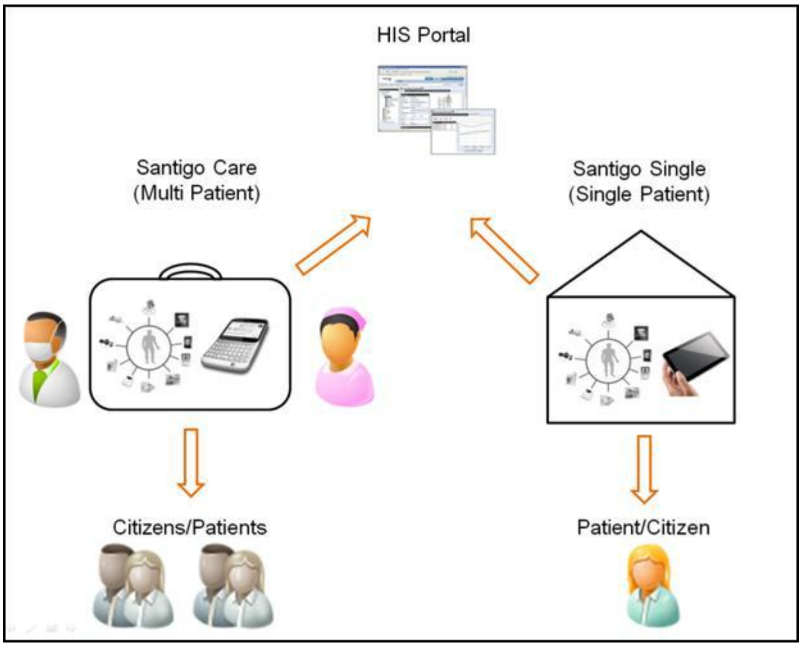

Figure 5: HIS Portal

Figure 6 below shows the technical architecture of the HIS Santigo Solution based on a tablet application.

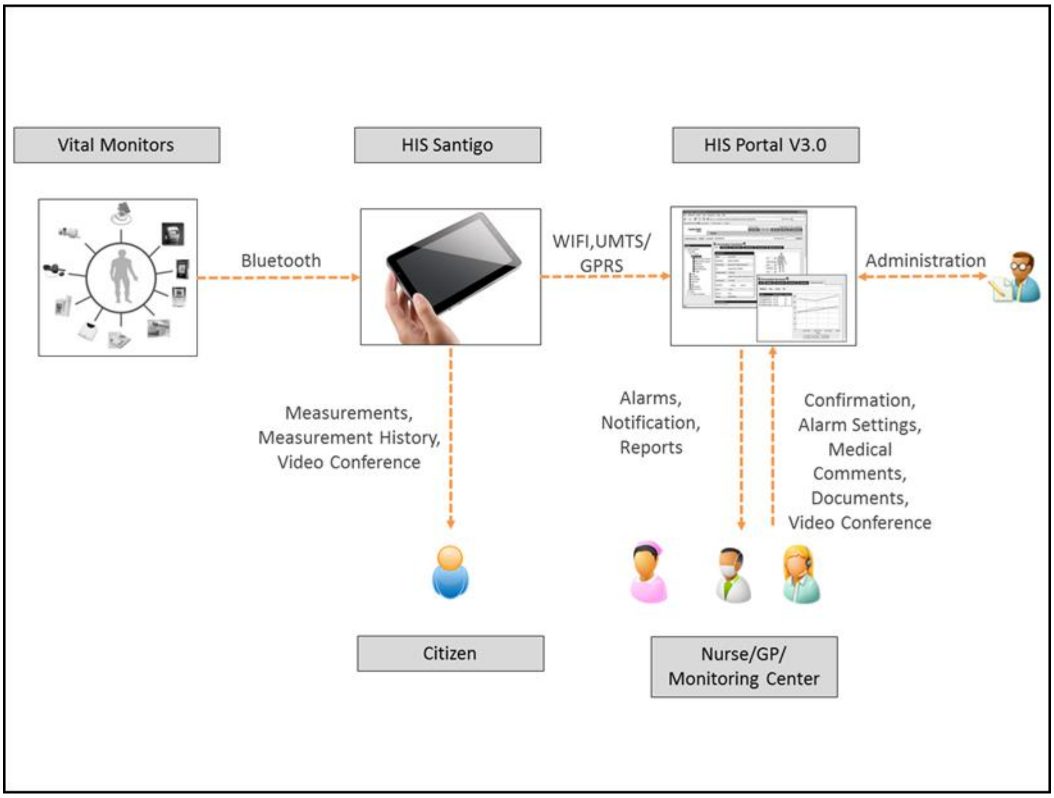

Figure 6: HIS Santiago Solution: technical architecture
