# Supplementary material for: BeyondSilos, a Telehealth-Enhanced Integrated Care Model in the Domiciliary Setting for Older Patients: Observational Prospective Cohort Study for Effectiveness and Cost-Effectiveness Assessments
Source: JMIR Med Inform. 2020 Oct 6;8(10):e20938. doi: 10.2196/20938 (PMC7576466; doi:10.2196/20938)
Supplement: Multimedia Appendix 3 [file medinform_v8i10e20938_app3.pdf]

Tables of costs

Intervention costs

Intervention one-off costs (per patient)	
**Time spent by professionals (overhead 18%)**	**187.40**
Time spent by Physician (service development, receiving training, adaptation)	40.63
Time spent by Nurse (service development, receiving training, adaptation)	112.06
Time spent by Social worker (service development, receiving training, adaptation)	5.49
Time spent by Physicians and Nurses on training provision to clients / patients	0.63
**Installation of the software for Physicians and Nurses**	**2.50**
**Cost of the set of devices and the software for clients/patients**	**1,079.00**
**TOTAL**	**1,268.89**
**Intervention recurring costs (per patient and year)**	
Telecommunication costs	144.00
Software costs	86.4
**TOTAL**	**230.40**

Healthcare costs (per patient and year in €)

Control Group baseline health	
**Total time spent by professionals in usual care (overhead 18%)**	**509.02**
Time spent by Physicians in usual care (assessment and care planning, consultations)	58.46
Time spent by Nurses in usual care (assessment and care planning, consultations)	291.06
Time spent by Social workers in usual care (care provision, home consultation) compared to BeyondSilos	81.86
**Travel costs in usual care (overhead 18%)**	**7.79**
Travel costs in usual care (service provider visits to the patient's home)	3.60
Travel costs of Social workers' trips in usual care compared to BeyondSilos	3
**Hospitalisation cost**	**4,681.81**
**TOTAL**	**5,198.62**
Control Group deteriorated health	
**Total time spent by professionals in usual care (overhead 18%)**	**509.02**
Time spent by Physicians in usual care (assessment and care planning, consultations)	58.46
Time spent by Nurses in usual care (assessment and care planning, consultations)	291.06
Time spent by Social workers in usual care (care provision, home consultation) compared to BeyondSilos	81.86
**Travel costs in usual care (overhead 18%)**	**7.79**
Travel costs in usual care (service provider visits to the patient's home)	3.60
Travel costs of Social workers' trips in usual care compared to BeyondSilos	3
**Hospitalisation cost**	**4,704.88**
**TOTAL**	**5,221.69**
Intervention Group baseline health	
**Total time spent by professionals in intervention (overhead 18%)**	**627.80**
Time spent by Physicians in intervention	292.30
Time spent by Nurses in intervention	207.90
Time spent by Social workers in intervention	31.83
**Hospitalisation cost**	**5,037.09**
**TOTAL**	**5,664.89**
Intervention Group deteriorated health	
**Total time spent by professionals in intervention (overhead 18%)**	**627.80**
Time spent by Physicians in intervention	292.30
Time spent by Nurses in intervention	207.90
Time spent by Social workers in intervention	31.83
**Hospitalisation cost**	**3,875.09**
**TOTAL**	**4,502.89**

Societal costs (per patient and year in €)

Control Group baseline health	
Extra travel time spent by patients in usual care compared to BeyondSilos	22.76
Extra travel costs and time for informal carers in usual care compared to BeyondSilos	37.76
Healthcare costs	5,198.62
**TOTAL**	**5,259.14**
Control Group deteriorated health	
Extra travel time spent by patients in usual care compared to BeyondSilos	22.76
Extra travel costs and time for informal carers in usual care compared to BeyondSilos	37.76
Healthcare costs	5,221.69
**TOTAL**	**5,282.21**
Intervention Group baseline health	
Time spent by patients using the service	288.26
Healthcare costs	5,664.89
**TOTAL**	**5,953.15**
Intervention Group deteriorated health	
Time spent by patients using the service	288.26
Healthcare costs	4,502.89
**TOTAL**	**4,791.15**
